# High-throughput RNA sequencing reveals distinct gene signatures in active IgG4-related disease

**DOI:** 10.1038/s41598-017-17602-9

**Published:** 2017-12-14

**Authors:** Brandon W. Higgs, Yanying Liu, Jianping Guo, Yinong Sebastian, Chris Morehouse, Wei Zhu, Limin Ren, Mengru Liu, Yan Du, Guangyan Yu, Lingli Dong, Hong Hua, Pan Wei, Yi Wang, Zhengang Wang, Yihong Yao, Zhan-Guo Li

**Affiliations:** 1 MedImmune, Gaithersburg, MD 20878 USA; 2 Department of Rheumatology and Immunology, Peking University People’s Hospital, Beijing, 100044 China; 3 Department of Oral and Maxillofacial Surgery, Peking University School of Stomatology, Beijing, 100081 China; 4 Department of Immunology and Rheumatology, Tongji Hospital, Tongji Medical College, Huazhong University of Science and Technology, Wuhan, 430000 China; 5 Department of Oral Medicine, Peking University School of Stomatology, Beijing, 100081 China; 6 Department of Radiology, Peking University People’s Hospital, Beijing, 100044 China; 7 Department of Rheumatology and Immunology, Beijing Tongren Hospital, Beijing, 1000730 China

## Abstract

We aimed to characterize the molecular differences and effects from prednisone treatment among IgG4-related disease with salivary gland lesions (RD-SG), without SG lesions (RD-nonSG), and IgG4-related retroperitoneal fibrosis (RF). RNA sequencing was conducted on blood from 25 RD-SG, 11 RD-nonSG, 3 RF and 10 control subjects. Among these, 8 RD-nonSG and 12 RD-SG patients were subjected to treatment with prednisone and/or glucocorticoid-sparing agents. Six RD patients had a longitudinal time point. The mRNA levels of *IgG4* and *IgE*, genes specific for Th2 cells, eosinophils, and neutrophils were over-expressed in RD-SG and RD-nonSG. A B-cell signature was suppressed in patients group versus controls, while Th1, Th2, Treg, and eosinophil gene signatures were increased in patients without treatment. Interestingly, Tfh genes and B cell signature were decreased at flare disease state. Prednisone treatment led to increased neutrophil, but decreased Treg signatures. Serum IgG4 levels correlated with the eosinophil and neutrophil gene signatures in RD-SG patients, and with a B cell signature in only RD-nonSG patients. IgG4, IgE, and cell-specific signatures are regulated in patients, suggesting the imbalance of immune and inflammatory cells in IgG4-related disease. Prednisone treatment selectively modulates Treg, eosinophil, and neutrophil signatures.

## Introduction

IgG4-related disease (IgG4-RD) is a systemic disorder involving a spectrum of multiple indications, distinguished by *often* elevated levels of serum IgG4, infiltration of IgG4^+^ plasma cells into target tissues, and diffuse swelling, mass formation, or fibrosis of affected organs^1^. This disease affects men approximately two-fold more often than women and age at diagnosis ranges from 50 to 70 years^2^. Most patients do respond to steroids initially, although relapse is observed in up to 47% cases^3–5^. Various histopathological features are shared among different IgG4-RD indications, which challenge diagnosis, although certain syndromes have organ-specific involvement^6^. Some examples include: Mikulicz’s disease affecting the salivary and lacrimal glands, autoimmune pancreatitis affecting the pancreas, Riedel’s thyroiditis affecting the thyroid, and Morbus Ormond or retroperitoneal fibrosis (RF), affecting tissue in the retroperitoneum, to name a few^2^.

Beyond the evidence of certain genetic risk factors^7–11^, IgG4-RD is mechanistically thought to be activated by the innate response to pathogens that mimic self-antigen, leading to an autoimmune response^2,6^. Type 1 helper T cells (Th1) are thought to support innate immune response to infection, which then shifts to type 2 helper T cells (Th2) involvement with increases in expression of IL-4, IL-5, and IL-13 mRNA and protein in both the affected tissue and peripheral compartments^12–15^. Th2 adaptive response can affect Th1 response, thus this Th1/Th2 balance is important in regulation^1^. Regulatory T cells (Tregs) are also activated, with accumulation of CD4^+^CD25^+^ T cell infiltrates and abundance of IL-10, FOXP3, and TGF-β1^12,16,17^. The increase of these cytokines promotes eosinophilia in the serum or tissue, high levels of IgG4-producing plasma cells, elevated production of IgE, and fibrosis, with inflammatory cell infiltrates ultimately causing organ damage^6^.

Recently, studies have utilized transcript profiling in labial salivary glands (LSGs) to identify distinguishing molecular features between IgG4-RD and Sjögren’s syndrome (SS), a disease with common phenotypic elements^18–20^. Among other findings, active involvement of Th2- (*IL-4, IL-5*, and *IL-21*), T follicular helper cell (Tfh)- (*BCL-6* and *CXCR5*) and T-reg- (*IL-10, FOXP3*, *CCL18*, and *TGF-β1*) related transcripts in patients with IgG4-RD was observed. These data showed how elevated levels of such cytokines and chemokines can induce IgG4 plasma cell infiltration, high IgG4 levels in the periphery, and impact tissue fibrosis in the LSG of IgG4-RD patients^19^. However, no studies to date have assessed the differences in molecular pathways or cell populations among IgG4-related disease with salivary gland lesions (RD-SG), without SG lesions (RD-nonSG), and IgG4-related retroperitoneal fibrosis (RF), in the peripheral blood, as well as the effects of corticosteroids on these signaling pathways.

In this study, we used whole transcriptomic sequencing to identify and distinguish both cell and pathway-associated activation in the blood of healthy subjects or those with RD-SG, RD-nonSG, or RF. A large cohort of patients was transcript profiled at a relative baseline time point, with two patients providing additional post baseline flare specimens. To better understand the possible mechanism(s) implicated in the treatments of IgG4-RD, we evaluated the effects of prednisone on the molecular pathways most relevant to disease activity. Additionally, cell-specific gene signatures linking the B and T cell axes were assessed to elucidate cellular involvement, as well as the correlation with *IgG4* mRNA levels across the three diseases.

## Results

### Transcriptome profiles in patients with RD-SG, RD-nonSG, or RF and healthy controls using principal components analysis

**Figure 1:**
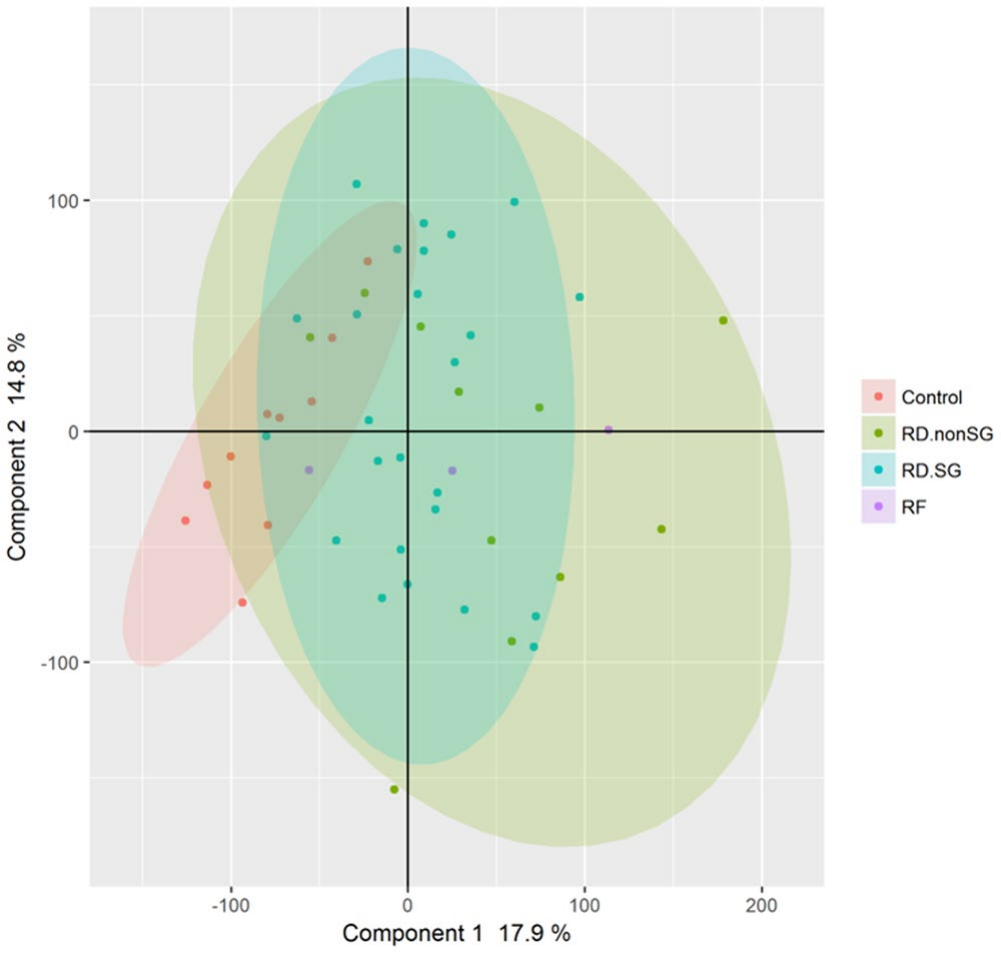
Principal components analysis plot of RD-SG, RD-nonSG, RF, and control subjects using the whole transcriptome.

Principal components analysis (PCA) was used to elucidate the whole transcriptome profile among the three diseases in relation to healthy subjects (Fig. 1). Though the plot displayed an overlap in disease and control cohorts, there was an apparent difference between the control subjects and disease subjects. Specifically, along the x-axis (principal component 1), controls (red) were the leftmost cohort, followed by the other disease groups. More relevant was the smaller within-disease variability that was apparent in the control and RD-SG (blue) compared to the RD-nonSG cohort (green). The RF cohort (purple) was very small (n = 3), thus the distribution of these points were difficult to interpret.

### *IgG4* and *IgE* are the most over-expressed transcripts in RD-SG and RD-nonSG patients, and suppressed by prednisone in RD-SG patients

**Figure 2:**
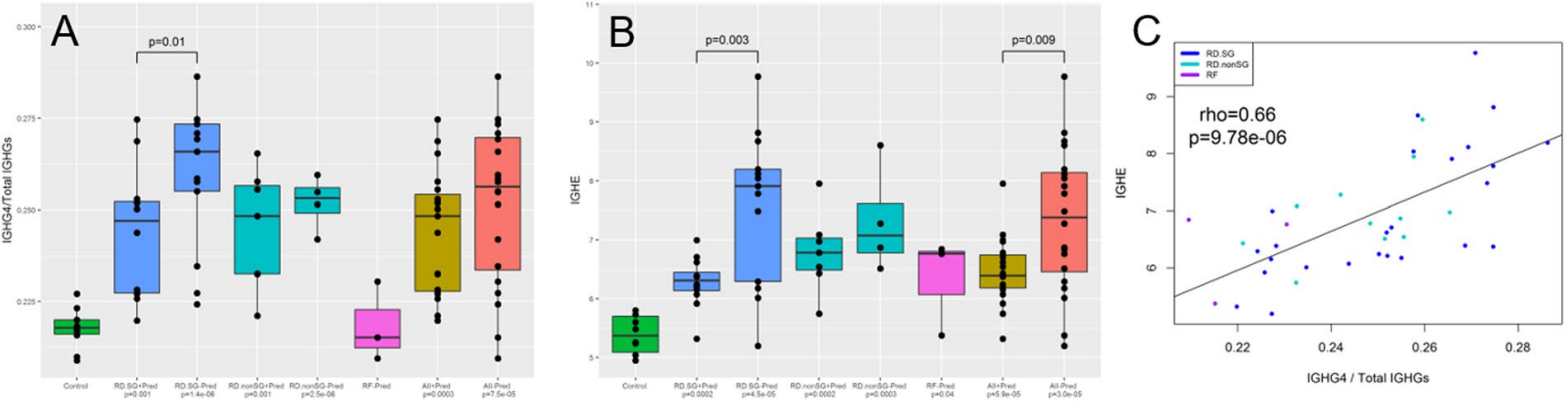
Distribution of *IgG4* and *IgE* mRNAs. (**A**) Expression of *IgG4* scaled by all *IgG1*, *IgG2*, and *IgG3* transcripts and (**B**) *IgE* across control subjects, RD-SG patients on prednisone treatment, RD-SG patients not on prednisone treatment, RD-nonSG patients on prednisone treatment, RD-nonSG patients not on prednisone treatment, RF patients, all patients on predisone treatment, and all patients not on prednisone treatment. (**C**) Correlation between *IgE* and *IgG4*/Total *IgG* mRNAs for all three diseases. P-values under each disease group indicate comparisons to control and are adjusted by age. Pred +  = currently treated with prednisone; Pred− = not currently treated with prednisone.

*IgG4* and *IgE* were identified as two of the most over-expressed transcripts in both RD-nonSG and RD-SG compared to the control cohort (Supplementary Table 1). These two cohorts were stratified by patients who were currently being treated with prednisone. All four patient cohorts had significantly higher mRNA expression of *IgG4*/*Total IgGs* and *IgE* (p ≤ 0.001 for all cohorts; Fig. 2A,B). RD-SG patients treated with prednisone had significant suppression of *IgG4*/*Total IgGs* (p = 0.01) and *IgE* (p = 0.003) mRNAs compared to those not treated, while RF patients showed difference in *IgE* from controls, though the sample size was small (p = 0.04). *IgG4*/*Total IgGs* and *IgE* mRNAs were highly correlated across the diseases (Fig. 2C; rho = 0.66, p < 9.78 × 10^−6^).

A linear model was constructed to identify genes across the transcriptome most correlated with *IgG4* mRNA levels. This approach was used to 1) adjust for transcripts modulated by prednisone treatment, and 2) distinguish transcripts unique to one of RD-SG, RD-nonSG, or RF cohorts. Among the top 50 positively and negatively correlated transcripts with *IgG4* (p < 0.01), 39/100 were associated with RD-SG (p < 0.01), 28/100 with RD-nonSG (p < 0.01), and 3 with RF (p < 0.01), with 23 being shared between RD-SG and RD-nonSG and 2 associated with RF shared with RD-SG and RD-nonSG cohorts (*CCL23* was unique to RF; Supplementary Table 2). This indicates that similar transcripts correlate with *IgG4* in all three diseases. Among these top 50 most positively correlated genes with *IgG4*, regardless of disease (and adjusting for prednisone treatment), the most activated biological categories were immunoglobulin (*IGHG1*, *IGHG3*, *IGHV1-69*, *IGHV3-30*, *IGKV3-20*, *IGKV3D-15*, *IGLV2-11*, and *IGLV3-21*), followed by nuclear division/mitosis/replication (*CDK1*, *CDC20*, *CDC6*, *DLGAP5*, *MCM10*, *RRM2*, *KIF4A*, and *TOP2A*).

### Treg, Th2, eosinophil, and neutrophil gene signatures are over-expressed in RD-SG and RD-nonSG, with a B cell signature suppressed in all diseases

**Figure 3:**
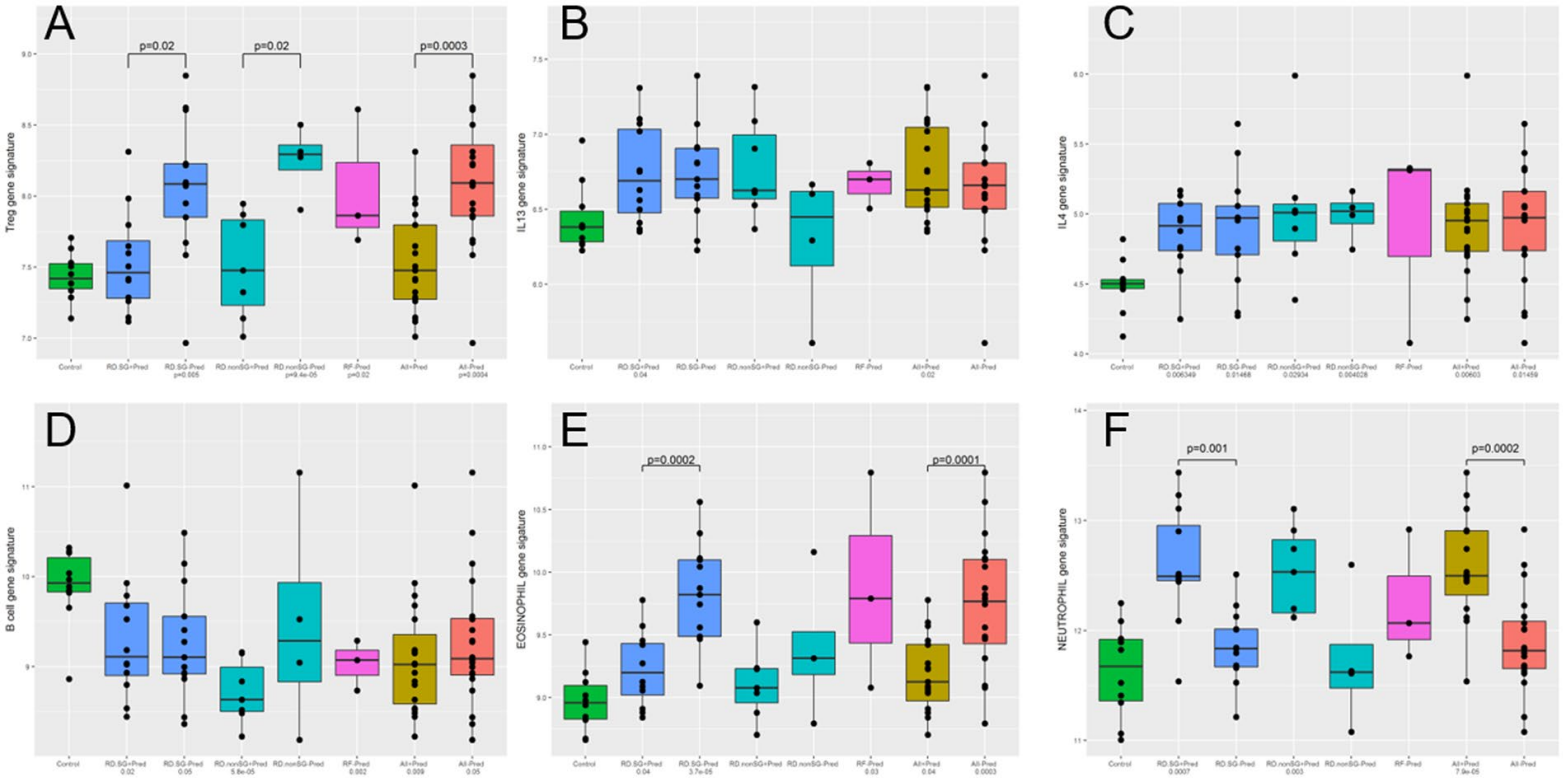
Distribution of cell-specific gene signatures. Expression of gene signatures of (**A**) Treg, (**B**) IL-13, (**C**) IL-4, (**D**) B cell, (**E**) eosinophil, and (**F**) neutrophil across control subjects, RD-SG patients on prednisone, RD-SG patients not on prednisone, RD-nonSG patients on prednisone, RD-nonSG patients not on prednisone, RF patients, all patients on prednisone combined, and all patients not on prednisone. P-values under each disease group indicate comparisons to control and are adjusted by age. Pred +  = currently treated with prednisone; Pred− = not currently treated with prednisone.

Various cell-specific gene signatures were used to evaluate cell population involvement in the diseases studied here. Interestingly, a Treg gene signature showed significant over-expression in RD-SG and RD-nonSG patients without treatment with prednisone compared to controls (Fig. 3A). Regarding the Th2 cytokine signature, while IL-13 gene showed significant over-expression in only RD-SG patients compared to controls regardless of prednisone treatment, IL-4 gene showed significant elevation in both RD-SG and RD-nonSG patients compared to controls (Fig. 3B and C). The B cell signature was significantly suppressed in the majority of patient cohorts (including RF) (Fig. 3D). The eosinophil and neutrophil gene signatures showed opposite effects between patients with or without prednisone treatment, respectively, in RD-SG. Specifically, compared to patients without prednisone treatment, the eosinophil gene signature was significantly suppressed (Fig. 3E, p = 0.0001 in RD-SG,), whereas the neutrophil gene signature was significantly elevated in RD-SG patients treated with prednisone (Fig. 3F, p = 0.01). Interestingly, a plasma cell gene signature showed no changes in any of the diseases with or without treatment, compared to controls (data not shown).

### A molecular characterization of cell-specific gene signatures in RD-SG, RD-nonSG, and RF patients and controls

**Figure 4:**
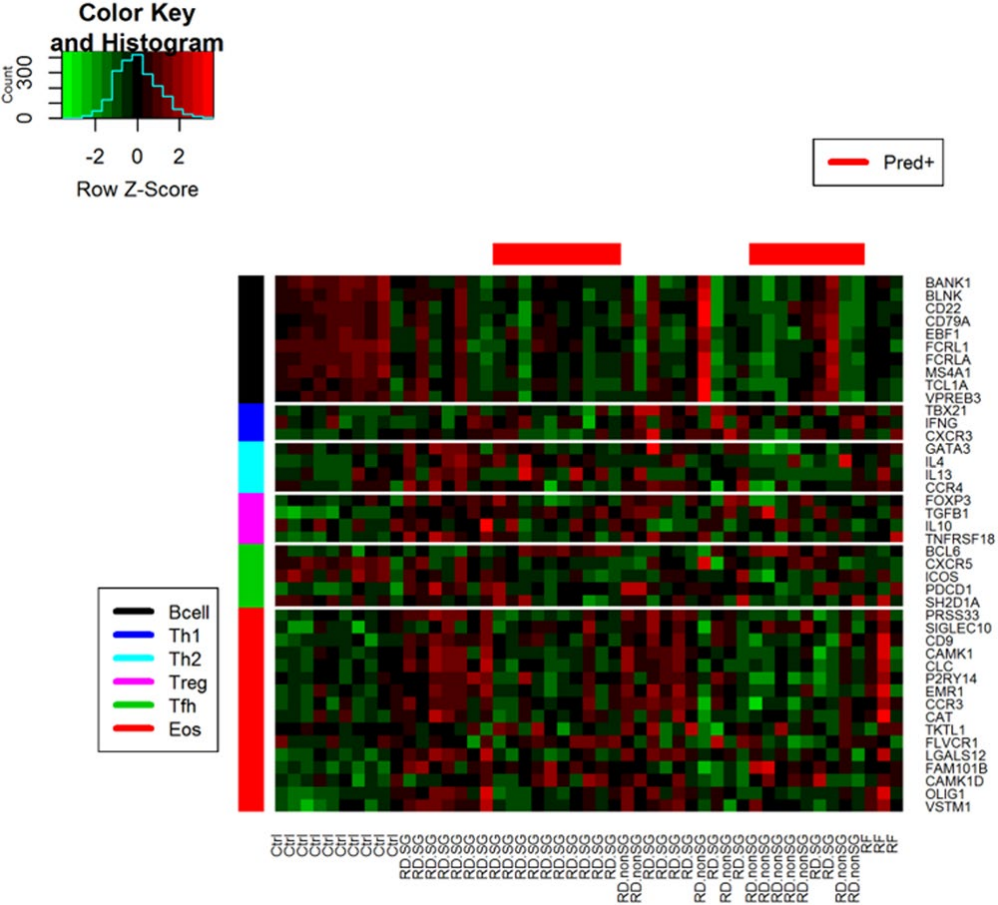
Heatmap of cell-specific gene signatures across the control, RD-SG, RD-nonSG, and RF cohorts. Ctrl = control subjects; Pred +  = currently treated with prednisone.

The molecular characterization of the cell-specific gene signatures, i.e. B, Th1, Th2, Treg, Tfh, and eosinophil cells, were analyzed across case and control cohorts (Fig. 4). From the heatmap, the effect of prednisone on all T cell sub-populations is evident. In general, the gene signatures were down-regulated in all patients treated with prednisone. The pattern seemed more apparent in T cell sub-populations of Th1, Treg, and Tfh in RD-SG patients. A similar pattern was seen in the eosinophil gene signature in both RD-SG and RD-nonSG patients. For the B cell signature, most genes were suppressed in RD-SG and RD-nonSG patients regardless of treatment, compared to healthy controls, though a few patients with active disease showed elevated expression across all genes (red vertical stripes in the B cell signature).

### *IgG4* and *IgE* mRNAs, T, B, and eosinophil cell-specific genes/gene signatures differed among RD-SG/RD-nonSG patients with flare or stable status

**Figure 5:**
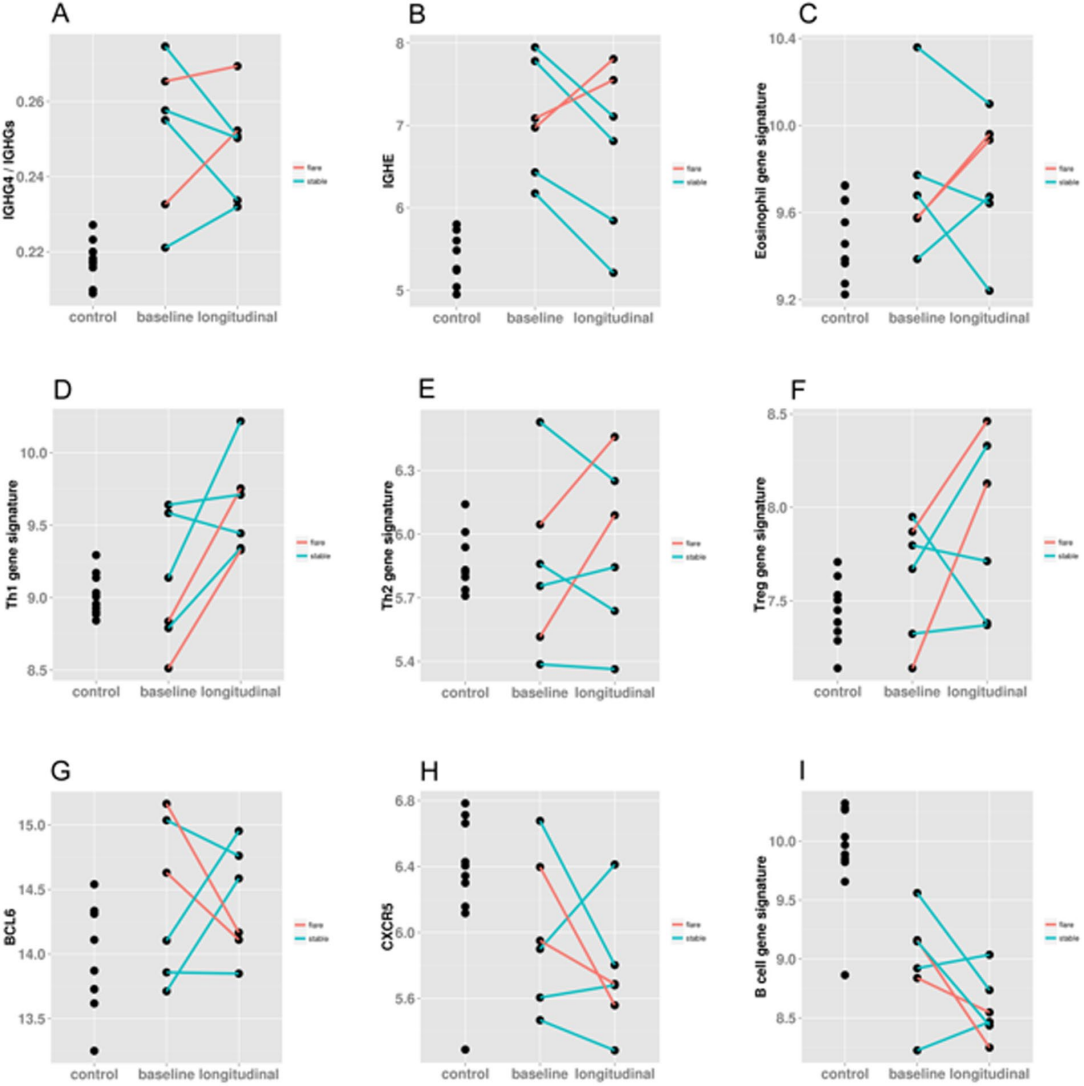
Longitudinal gene or gene signature profiles of two patients at relative baseline and post relative baseline visits when a flare was observed. All plots include the control cohort and all the 6 patients for (**A**) *IgG4*/Total *IgG*, (**B**) *IgE*, (**C**) eosinophil gene signature, (**D**) Th1 gene signature, (**E**) Th2 gene signature, (**F**) Treg gene signature, (**G**) BCL6, (**H**) CXCR5, and (**I**) B cell gene signature. Pink = patient experienced flare at visit 2; Blue = patient had stable disease at visit 2.

In addition to a baseline time point, blood was procured at a second time point from six RD-SG/RD-nonSG patients, two of whom experienced a flare. Though the exact time differences between the relative baseline and post baseline visit were not identical for each patient, the general molecular patterns were consistent for the two flare patients and differed for the four stable patients at the second visit (Fig. 5). For each RD-SG/RD-nonSG patient, there was induction at the flare time point in Th1, Th2, Treg, and eosinophil gene signatures. Similar induction at the flare time point was observed in *IgG4* and *IgE* mRNA levels (Fig. 5A–F). In contrast, the B cell signature, as well as two genes associated with Tfh cells (*BCL6* and *CXCR5*), all showed suppressed profiles at the flare time point(Fig. 5G–I). The control cohort was provided in each plot to indicate relative similarity of expression levels to a normal healthy population.

### IgG4 serum levels correlate with cell signatures

**Figure 6:**
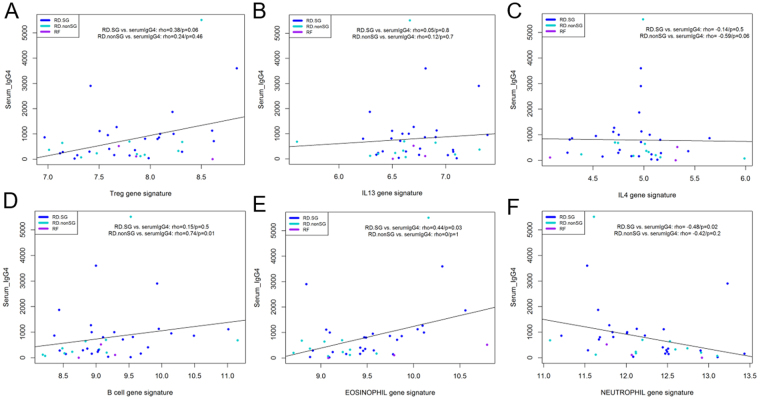
Correlation between serum levels of IgG4 and (**A**) Treg signature, (**B**) IL13 signature, (**C**) IL4 signature, (**D**) B cell signature, (**E**) eosinophil signature, (**F**) neutrophil signature in RD-SG, RD-nonSG, and RF patients.

Serum levels were correlated with cytokine and cell signatures described previously in RD-SG and RD-nonSG patients. IgG4 correlated with eosinophil and neutrophil gene signatures (rho = 0.44, p = 0.03 and rho = −0.48, p = 0.02, respectively) in RD-SG patients. In RD-nonSG, IgG4 levels correlated only with a B cell signature (rho = 0.74, p = 0.01) (Fig. 6). No other associations between serum levels of IgG4 and gene signatures were observed.

## Discussion

We used RNA sequencing to molecularly profile a large cohort of RD-SG, RD-nonSG, and RF patients. We showed that *IgG4* and *IgE* are among the most expressed transcripts in the blood of RD-SG or RD-nonSG patients, though not RF compared to controls, and both genes are highly correlated with each other. We also demonstrate that prednisone suppresses the levels of these genes in the blood in RD-SG, but not RD-nonSG patients. Reduction in serum IgG4 protein following steroid therapy has been observed previously in various studies as a result of immune suppression; glucocorticoid treatment is a well-known regimen to help attain remission in MD patients^21,22^. Among the 25 RD-SG patients, 17 of them in this study only have sialadenitis and dacryoadenitis involvement, which may explain the homogeneity of dramatic reduction in both *IgG4* and *IgE* levels in the blood from prednisone treatment. The higher intra-cohort variability observed in RD-nonSG compared to RD-SG patients in PCA across the whole transcriptome may also support this finding. Th2 gene signatures were generally increased in both RD-SG and RD-nonSG patients, and Treg gene signature was significantly reduced in patients treated with prednisone. Activation of Th2 cytokines and blood eosinophilia in RD patients has been previously reported, suggesting an allergy response mechanism in IgG4-RD, and eosinophilia is often treated with corticosteroids to promote cell death and clearance^23–25^. The increased neutrophil signature in RD-SG patients treated with prednisone may be explained by the well-known phenomena of glucocorticoid-induced granulocytosis, where leukocytes have increased release from the bone marrow, and reduced migration out of the circulation^26^. Within IgG4-RD specifically, a microarray study observed neutrophil-specific genes (DEFA3 and DEFA4) significantly over-expressed in peripheral blood mononuclear cells (PBMCs) of patients on steroid therapy compared to those not^27^.

Genes associated with mitosis, cell cycle, and replication were most correlated with *IgG4* expression. A previous study evaluating circulating autoantibodies in sera from IgG4-RD patients identified high levels of antibodies against prohibitin in patient subsets of autoimmune pancreatitis, MD, RF, IgG4-RD, and Sjögren’s syndrome (not healthy donors)^28^. The prevalence of anti-prohibitin auto-antibodies in IgG4-RD patients was hypothesized to increase cell proliferation, ultimately driving tissue enlargement. Additionally, a microarray study evaluating labial salivary glands in RD patients identified regulation of cell proliferation among the top enriched biological categories^19^. As increased IgG4 is a hallmark of this disease, the association between the phenotype and cell cycle processes is supported by these previous studies.

For RD patients with a longitudinal time point, there was an association between two patients that flared at the second visit (both on prednisone) and induction of Th1, Th2, Treg, and eosinophil gene signatures as well as *IgG4* and *IgE* mRNAs. In contrast, this pattern across these gene signatures/genes was not consistently observed in the four patients with stable disease status at the second visit. That is to say, no stable patient had multiple induced Th1, Th2, Treg, or eosinophil gene signatures at the second time point. The balance between cell-mediated immunity (Th1 cells), humoral immunity (Th2 cells), and maintenance of immune homoeostasis (Tregs) and how this correlates with disease pathogenesis or activity have been investigated in rheumatoid arthritis and SLE, though conclusions have varied^29,30^. Immune-activated over-expression of IL-4, -5, -10, -13, and TGF-β1 drives eosinophilia and increased IgG4 and IgE levels in IgG4-RD, thus this suggests that at states of increased disease activity, Th1, Th2, Treg, and eosinophil involvement would be greater^6^.

An inverse relationship was observed between the flare visit and both the B cell signature and Tfh genes, where the gene signature/genes were suppressed at the flare visit. Similar to those gene signatures/genes showing induction at the flare visit, there was no consistent pattern of agreement in the four patients with stable disease across these gene signatures/genes at the second time point. At baseline, the B cell signature was significantly reduced in all patients compared to controls and was even more pronounced at the longitudinal flare time point in the two patients, suggesting cell infiltration to the disease tissue from the periphery in increased disease activity states. Tfh cells are located within germinal centers and secrete IL-21, driving differentiation of B cells to produce antibodies, thus the pattern showed by these Tfh-associated genes at the flare time point is consistent with that of the B cell signature^31^. A study in PBMCs of SLE patients showed that flares may be positively correlated with expansion of both Tfh and regulatory B cells through a regulatory feedback mechanism^31^. Another study in SLE found that a peripheral subset of CD27-IgD-CD97 + memory B cells were increased with disease flare, though the entire subset of CD27-IgD- B cells had no correlation with disease activity^32^. These results are in contrast to the pattern observed in this study at the flare visit in the two patients, though the B cell signature used here is not specific to either regulatory or memory B cells, and as indicated in the study by Jacobi *et al*.^32^, differences in B cell subsets can greatly vary with respect to phenotype.

In summary, we show the importance of the T and B cell axis with molecular profiling across RD-SG, RD-nonSG, and RF as well as features that distinguish these three diseases. Future work seeks to better understand the molecular mechanisms at relapse or recurrence following steroid reduction in these patients.

## Methods

39 patients fulfilled the 2011 comprehensive IgG4-RD diagnostic criteria were involved in this study^33^. Among them, 26 patients were classified as definite IgG4-RD, 6 patients were classified as probable IgG4-RD and 7 patients were classified as possible IgG4-RD. Blood was procured from 25 RD-SG (ages 32–81; 12 Males), 11 RD-nonSG (ages 48–80; 9 Males), 3 RF (ages 48–65; 3 Males) and 10 control (ages 30–57; 7 Males) Chinese subjects (Table 1

**Table 1:** Subject summary: Control, RD-SG, RD-nonSG, and RF.

**No**.	**Sex**	**Age**	**organ involvement**												
			**sialad-enitis + dacryoa-denitis**	**eye**	**nose**	**deep lymph nodes**	**kidney**	**pancr-eatitis**	**liver**	**chola-ngitis**	**pro-statitis**	**thyroid**	**hypo-physis**	**retroper-itoneal fibrosis**	**inflam-matory pseudo-tumor**
**C1**	**F**	**38**													
**C2**	**M**	**31**													
**C3**	**M**	**30**													
**C4**	**M**	**48**													
**C5**	**F**	**52**													
**C6**	**M**	**32**													
**C7**	**M**	**49**													
**C8**	**M**	**57**													
**C9**	**F**	**48**													
**C10**	**M**	**30**													
**RD-SG1**	**M**	**55**	**X**												
**RD-SG2**	**M**	**65**	**X**												
**RD-SG3**	**M**	**44**	**X**												
**RD-SG4**	**F**	**54**	**X**												
**RD-SG5**	**M**	**55**	**X**												
**RD-SG6**	**F**	**50**	**X**												
**RD-SG7**	**M**	**56**	**X**												
**RD-SG8**	**F**	**41**	**X**												
**RD-SG9**	**M**	**54**	**X**												
**RD-SG10**	**F**	**49**	**X**												
**RD-SG11**	**M**	**32**	**X**												
**RD-SG12**	**F**	**51**	**X**												
**RD-SG13**	**F**	**81**	**X**												
**RD-SG14**	**F**	**56**	**X**												
**RD-SG15**	**F**	**34**	**X**												
**RD-SG16**	**M**	**64**	**X**												
**RD-SG17**	**F**	**55**	**X**												
**RD-SG18**	**F**	**52**	**X**					**X**							
**RD-SG19**	**M**	**59**	**X**				**X**	**X**		**X**					
**RD-SG20**	**M**	**49**	**X**					**X**							
**RD-SG21**	**M**	**73**	**X**											**X**	
**RD-SG22**	**M**	**61**	**X**			**X**									
**RD-SG23**	**F**	**56**	**X**			**X**		**X**							
**RD-SG24**	**F**	**60**	**X**			**X**	**X**	**X**				**X**		**X**	
**RD-SG25**	**F**	**49**	**X**					**X**							
**RD-non SG1**	**M**	**48**		**X**	**X**	**X**									
**RD-non SG2**	**M**	**59**					**X**							**X**	
**RD-non SG3**	**M**	**56**						**X**		**X**					
**RD-non SG4**	**M**	**66**						**X**						**X**	
**RD-non SG5**	**M**	**80**								**X**	**X**			**X**	
**RD-non SG6**	**M**	**62**					**X**	**X**		**X**	**X**				
**RD-non SG7**	**M**	**55**					**X**	**X**			**X**	**X**	**X**		
**RD-non SG8**	**F**	**68**						**X**	**X**						
**RD-non SG9**	**M**	**58**						**X**		**X**					**X**
**RD-non SG10**	**M**	**73**						**X**		**X**					
**RD-non SG11**	**F**	**55**				**X**	**X**								
**RF-1**	**M**	**65**												**X**	
**RF-2**	**M**	**54**												**X**	
**RF-3**	**M**	**48**												**X**	
**No**.	**serum IgG4 (mg/dl)**	**pathology**		**treatment**							**Longi-tudinal**				
		**number of IgG4** ^**+**^ **plasma cell/HP**	**IgG4** ^**+**^ **plasma cell/IgG** ^**+**^ **plasma cell(%)**	**Pre-disone**	**Cyclopho-sphamide**	**Ursodes-oxycholic acid**	**Azathi-oprine**	**Tam-oxifen**	**Hydroxy-chloroquine**	**Metho-trexate**					
**C1**															
**C2**															
**C3**															
**C4**															
**C5**															
**C6**															
**C7**															
**C8**															
**C9**															
**C10**															
**RD-SG1**	**288**	**>10**	**>40**	**5 mg qd**	**0.6/2 m**										
**RD-SG2**	**35.2**	**>10**	**>40**	**5 mg qd**					**0.2 bid**						
**RD-SG3**	**281**	**>10**	**>40**	**5 mg qd**	**0.4 /m**										
**RD-SG4**	**402**	**>10**	**>40**	**5 mg qd**	**0.4/2 m**										
**RD-SG5**	**710**	**>10**	**>40**												
**RD-SG6**	**948**	**>10**	**>40**												
**RD-SG7**	**3600**	**>30**	**>40**												
**RD-SG8**	**18.5**	**>10**	**50**	**15 mg qd**											
**RD-SG9**	**230**	**ND**	**ND**	**7.5 mg qod**						**7.5 mg qw**					
**RD-SG10**	**159**	**>10**	**>40**	**5 mg qd**	**0.4 /m**										
**RD-SG11**	**1000**	**100**	**50**												
**RD-SG12**	**859**	**100**	**>40**												
**RD-SG13**	**357**	**>10**	**>50**												
**RD-SG14**	**1870**	**>10**	**>40**												
**RD-SG15**	**145**	**>10**	**>40**	**5 mg qd**											
**RD-SG16**	**155**	**40**	**50**	**2.5 mg qd**											
**RD-SG17**	**296**	**ND**	**ND**	**2.5 mg qd**					**0.2 bid**						
**RD-SG18**	**1130**	**ND**	**ND**												
**RD-SG19**	**800**	**>10**	**>40**												
**RD-SG20**	**1270**	**ND**	**ND**								**X**				
**RD-SG21**	**807**	**>10**	**>40**								**X**				
**RD-SG22**	**2900**	**100**	**>40**	**30 mg qd**											
**RD-SG23**	**1110**	**ND**	**ND**	**60 mg qd**					**0.2 bid**						
**RD-SG24**	**864**	**>10**	**>40**												
**RD-SG25**	**1001**	**>10**	**>40**												
**RD-non SG1**	**5510**	**>50**	**50**												
**RD-non SG2**	**117**	**>50**	**>40**												
**RD-non SG3**	**364**	**>10**	**>40**	**20 mg qd**		**250 mg tid**									
**RD-non SG4**	**69.9**	**>10**	**>40**	**10 mg qd**	**0.4/2w**						**X**				
**RD-non SG5**	**642**	**ND**	**ND**	**2.5 mg qd**		**250 mg tid**	**50 mg qd**	**10 mg bid**			**X**				
**RD-non SG6**	**200**	**>10**	**>40**	**5 mg qd**							**X**				
**RD-non SG7**	**697**	**50**	**100**	**20/17.5 mg qod**			**50 mg qd**				**X**				
**RD-non SG8**	**680**	**ND**	**ND**												
**RD-non SG9**	**172**	**>30**	**>40**	**2.5 mg qd**											
**RD-non SG10**	**230**	**>10**	**>40**	**10 mg qod**											
**RD-non SG11**	**324**	**>10**	**>40**				**50 mg qod**								
**RF-1**	**105**	**>10**	**>40**												
**RF-2**	**520**	**>10**	**>40**												
**RF-3**	**<0.357**	**>10**	**>40**												

). Any organ with the salivary and lacrimal gland was involved in RD-SG patients. Except the salivary and lacrimal gland, other organs were involved in RD-nonSG patients. However, only retroperitoneal fibrosis was found in RF patients. Twenty patients were treated with prednisone (≤ 60 mg), with or without another glucocorticoid-sparing agent (cyclophosphamide, ursodeoxycholic acid, azathioprine, tamoxifen, hydroxychloroquine, methotrexate, and/or mycophenolate mofetil). Six patients had one longitudinal time point: two patients exhibited a flare at this second time point, while four did not. All participants provided written informed consent, in accordance with the Declaration of Helsinki. The study was approved by the Ethical Committee of Peking University People’s Hospital.

In this study, stable or active condition was defined for every subject at the first visit. Stable condition was defined as the disappearance of clinical symptoms, normalization or stabilization of serum IgG or IgG4, and resolution of organ manifestations on imaging. Or else, it was defined as active condition. At the longitudinal time point, we defined flare condition as a recurrence of symptoms with the development or reappearance of organ involvement or abnormalities on imaging studies and elevation of serum IgG or IgG4 level.

The IL-13 and IL-4 gene signatures were identified as what have been described previously^34,35^. The B and plasma cell gene signatures were developed using experiments as described in Streicher *et al*.^36^. The eosinophil and neutrophil gene signatures were identified from a phase 1 clinical trial in systemic lupus erythematous (SLE)^37^. Baseline blood cell counts of SLE patients were correlated with whole genome microarray transcript profiles measured in the blood of the same patients. Th1, Th2, Treg, and Tfh gene signatures were taken from Dong *et al*.^38^. The genes that compose each gene signature are provided in Supplementary Table 3.

The methods for RNA sequence read mapping and differential expression analysis are provided in the Supplementary Methods.

## Electronic supplementary material

Supplementary information
