# Supplementary material for: High-throughput RNA sequencing reveals distinct gene signatures in active IgG4-related disease
Source: Sci Rep. 2017 Dec 14;7:17567. doi: 10.1038/s41598-017-17602-9 (PMC5730556; doi:10.1038/s41598-017-17602-9)
Supplement: Supplementary file 1 — Supplementary information [file 41598_2017_17602_MOESM1_ESM.doc]

**High-throughput RNA sequencing reveals distinct gene signatures in active IgG4-related disease**

Brandon W. Higgs^a, 1^, Yanying Liu^b, 1^, Jianping Guo^b, 1^, Yinong Sebastian^a^, Chris Morehouse^a^, Wei Zhu^a^, Limin Ren^b^, Mengru Liu^b^, Yan Du^b^, Guangyan Yu^c^, Lingli Dong^d^, Hong Hua^e^, Pan Wei^e^, Yi Wang^f^, Zhengang Wang^g^, Yihong Yao^a±^, Zhan-Guo Li^b±^

**Supplementary Methods**

**RNA sequence read mapping and differential expression analysis**

RNA sequencing data (RNASeq) data was generated using the Illumina standard library preparation and sequencing protocols as described in (Guo et al, 2013). Paired end FASTQ files of 90mer sequence reads were QCd for read counts, quality values, kmer usage, GC-content, and all other relevant parameters with FastQC (v0.10.1). The average read count per mate was 50 million. RNA reads were mapped to the human genome (UCSC hg19; Feb 2009 release; Genome Reference Consortium GRCh37) using TopHat2 (v2.0.9; Kim et al, 2013) and the human reference gtf annotation file (GRCh37.68). Transcript counts were calculated and normalized using htseq-count and DESeq (v1.12.1; Anders et al, 2010). To adjust for age-related differences between control and disease subjects, a linear model was conducted for all case/control comparisons and all p-values were adjusted by age.

A multiple regression model was used to identify genes most correlated with *IgG4* across the three diseases and healthy subjects, adjusting for steroid use. The independent variables included RD-SG status, RD-nonSG status, RF status, prednisone use, and *IgG4* levels, all predicting each transcript as individual models. All calculations and figures were created in R (v3.1.0; R: A Language and Environment for Statistical Computing, R Development Core Team, R Foundation for Statistical Computing, Vienna, Austria, 2014). Pathway analyses were conducted with DAVID (Huang et al, 2009).

**References**

Guo G, Sun X, Chen C, Wu S, Huang P, Li Z et al., Whole-genome and whole-exome sequencing of bladder cancer identifies frequent alterations in genes involved in sister chromatid cohesion and segregation. Nat Genet. 45(12):1459-63 (2013).

Kim D, Pertea G, Trapnell C, Pimentel H, Kelley R, Salzberg SL. TopHat2: accurate alignment of transcriptomes in the presence of insertions, deletions and gene fusions. Genome Biol. 2013 Apr 25;14(4):R36. doi: 10.1186/gb-2013-14-4-r36.

Anders S, Huber W: Differential expression analysis for sequence count data Genome Biology 11 11(10):R106 (2010).

Huang DW, Sherman BT, Lempicki RA. Systematic and integrative analysis of large gene lists using DAVID Bioinformatics Resources. Nature Protoc. 2009;4(1):44-57.

| **Supplementary Table 1. Top over/under expressed genes in RD-SG and RD-nonSG cohorts** | | | | | | | |
| --- | --- | --- | --- | --- | --- | --- | --- |
| **Up-regulated genes** | | | | **Down-regulated genes** | | | |
| **SG.pred-** | **SG.pred+** | **nonSG.pred-** | **nonSG.pred+** | **SG.pred-** | **SG.pred+** | **nonSG.pred-** | **nonSG.pred+** |
| IGHG4 | HLA-P | IGHG4 | ADAMTS2 | LRRN3 | IQSEC3 | GSTM1 | XIST |
| IGHE | ADAMTS2 | HLA-DRB5 | DAAM2 | RP11-54O7.1 | RP11-54O7.1 | LRRN3 | CNTNAP2 |
| HLA-DRB5 | OLAH | KRT1 | DEFA4 | SLC4A10 | IGLV3-12 | RP11-54O7.1 | SLC4A10 |
| NRIP3 | DAAM2 | GPX1P1 | CA1 | CNTNAP2 | DSP | ZBED6 | PAX5 |
| HDC | CD177 | IGHE | CD177 | NRCAM | LRRN3 | ZNF208 | FCRL1 |
| CCL23 | IGHG4 | HBM | ACTBP8 | DSC1 | LCN8 | CLEC4F | hsa-mir-4537 |
| CYP2B6 | ARG1 | AC079949.1 | DEFA1 | DSP | CNTNAP2 | COL4A3 | DSP |
| EDN3 | AC005392.1 | TMPRSS9 | DEFA1B | SLC38A11 | USP9Y | NRCAM | LRRN3 |
| CACNG6 | IL1R2 | PCSK1N | S100P | USP9Y | ZNF208 | SLC38A11 | NRCAM |
| CDO1 | ECHDC3 | ACTBP8 | OLAH | DDX3Y | CTD-2611K5.6 | AC092214.10 | COL19A1 |
| PRSS33 | CA1 | FTH1 | TUBB2A | CA6 | COL19A1 | PHIP | PTPRK |
| RP11-731F5.1 | TUBB2A | HLA-G | ELANE | NPAS2 | NRCAM | SLC4A10 | IGHJ4 |
| CLC | ALOX15B | RP1-286D6.2 | ORM1 | IQSEC3 | CYorf15B | CNTNAP2 | CR2 |
| TNFRSF4 | TPST1 | C4orf48 | PRTN3 | ZFY | RP11-1021N1.2 | RP11-17A4.2 | IGHJ5 |
| ADCY1 | CEBPD | CCDC85B | DEFA3 | COL4A3 | SLC4A10 | NOG | KLHL14 |
| BEGAIN | FTH1 | ANKRD9 | AZU1 | NOG | KDM5D | AC104135.3 | SLC38A11 |
| PTGDR2 | GPX1P1 | TPGS1 | PGLYRP1 | CYorf15B | DDX3Y | RP11-90P13.1 | FCRL2 |
| GATA2 | HLA-G | HLA-DRB6 | RP11-20D14.6 | CCR12P | ZFY | C1orf132 | AL122127.3 |
| RSAD2 | INHBB | FAM108A6P | CTSG | COL19A1 | CTD-2215E18.1 | WWC2 | AL122127.1 |
| HES4 | AC079949.1 | HBQ1 | CBS | PRSS35 | SLC38A11 | KLHL11 | IGHJ6 |

| **Supplementary Table 2. Top 50 positively and top 50 negatively correlated transcripts with IgG4 mRNA using a linear model** | | | | | | | | | | | | | | | | |
| --- | --- | --- | --- | --- | --- | --- | --- | --- | --- | --- | --- | --- | --- | --- | --- | --- |
| ensembl | gene.name | intercept | intercept pv | RD.nonSG coefficient | RD.nonSG pv | RD.SG coefficient | RD.SG pv | RF coefficient | RF pv | IgG4 coefficient | IgG4 pv | predisone coefficient | predisone pv | adjusted r^2 | r^2 | IgG4 correlation |
| ENSG00000211892 | IGHG4 | 3.39296E-15 | 2.08378E-06 | 7.85194E-16 | 0.017833744 | 9.44843E-16 | 0.002696047 | 1.46168E-16 | 0.678844194 | 1 | 0 | -2.36427E-17 | 0.907011744 | 1 | 1 | positive |
| ENSG00000211891 | IGHE | 0.44978666 | 0.458378956 | 0.721199255 | 0.024496835 | 0.391765389 | 0.180788144 | 0.760346052 | 0.030725835 | 0.54481929 | 8.05967E-11 | -0.31294509 | 0.116406731 | 0.788369704 | 0.810414526 | positive |
| ENSG00000211662 | IGLV3-21 | 3.303002916 | 0.000373871 | -0.526044672 | 0.238622418 | -0.980150086 | 0.021199006 | -0.231555807 | 0.634952209 | 0.471952906 | 5.17936E-06 | 0.127276945 | 0.649264334 | 0.379396256 | 0.444042479 | positive |
| ENSG00000211897 | IGHG3 | 5.204731214 | 6.03318E-10 | -0.321281477 | 0.347076518 | -0.308446654 | 0.3323262 | 0.2701234 | 0.471422636 | 0.454227772 | 6.32425E-08 | -0.113641315 | 0.59702151 | 0.575769095 | 0.619959814 | positive |
| ENSG00000239975 | IGKV1D-33 | 3.066188839 | 1.93026E-06 | -0.535368848 | 0.068792213 | -0.45178602 | 0.097855852 | -0.034143125 | 0.914334225 | 0.453359733 | 1.38937E-09 | -0.227568829 | 0.215626745 | 0.659087781 | 0.694599471 | positive |
| ENSG00000242076 | IGKV1-33 | 3.066831961 | 1.88137E-06 | -0.534838016 | 0.068739053 | -0.450508426 | 0.098389824 | -0.033129566 | 0.916768845 | 0.453183789 | 1.36148E-09 | -0.227632355 | 0.214964084 | 0.659586369 | 0.695046122 | positive |
| ENSG00000242371 | IGKV1-39 | 4.504624046 | 6.18072E-12 | -0.549298809 | 0.031907556 | -0.53630475 | 0.024796767 | -0.240889227 | 0.381578007 | 0.432192586 | 1.03706E-10 | -0.160395103 | 0.310746979 | 0.691110221 | 0.723286239 | positive |
| ENSG00000251546 | IGKV1D-39 | 4.51619429 | 7.15135E-12 | -0.540544973 | 0.035908104 | -0.53250614 | 0.026852366 | -0.234395591 | 0.397980574 | 0.429310404 | 1.52645E-10 | -0.156586558 | 0.325864722 | 0.68417993 | 0.717077854 | positive |
| ENSG00000211973 | IGHV1-69 | 3.129505655 | 0.000110131 | -0.787706134 | 0.043310647 | -0.52374497 | 0.144241039 | -0.448239619 | 0.287467021 | 0.428251781 | 1.97794E-06 | 0.182220157 | 0.449653244 | 0.455189059 | 0.511940198 | positive |
| ENSG00000243290 | IGKV1-12 | 3.108480389 | 9.81601E-05 | -0.725379128 | 0.058085723 | -0.844367056 | 0.01912858 | -0.259639966 | 0.529770065 | 0.411475323 | 3.08453E-06 | 0.063020565 | 0.79002933 | 0.390038267 | 0.453575948 | positive |
| ENSG00000197408 | CYP2B6 | -1.883595795 | 0.054540367 | -0.335722979 | 0.497338534 | 0.186922365 | 0.684269212 | 0.603696766 | 0.269415313 | 0.396412263 | 0.000310435 | -0.357015164 | 0.255287069 | 0.417248415 | 0.477951705 | positive |
| ENSG00000211940 | IGHV3-9 | 3.162534695 | 0.00017662 | -0.37627705 | 0.347791339 | -0.307284709 | 0.409566649 | 0.332585306 | 0.449810854 | 0.393738084 | 1.81429E-05 | -0.006251491 | 0.980185575 | 0.394043251 | 0.457163746 | positive |
| ENSG00000211673 | IGLV3-1 | 4.508997781 | 2.88123E-07 | -0.845449835 | 0.032336804 | -0.777084669 | 0.034461017 | 0.077368973 | 0.854880508 | 0.391205025 | 1.12547E-05 | 0.025504116 | 0.916319135 | 0.359427162 | 0.426153499 | positive |
| ENSG00000211896 | IGHG1 | 8.919480968 | 3.32326E-16 | -0.636570208 | 0.084123528 | -0.509695783 | 0.135599074 | -0.060673566 | 0.878959865 | 0.390343525 | 4.26654E-06 | 0.097798633 | 0.669130977 | 0.408282364 | 0.469919617 | positive |
| ENSG00000129596 | CDO1 | 0.324775648 | 0.736574565 | -0.149470866 | 0.763520782 | 0.261838318 | 0.571788317 | 0.603675287 | 0.272501857 | 0.387710489 | 0.000434744 | -0.28429183 | 0.366762399 | 0.396834552 | 0.459664287 | positive |
| ENSG00000115884 | SDC1 | -0.068279699 | 0.895154193 | -0.127523223 | 0.632895814 | -0.138437612 | 0.577677489 | 0.369164217 | 0.212345111 | 0.387261855 | 9.5059E-09 | -0.113377752 | 0.501718185 | 0.637933722 | 0.675648959 | positive |
| ENSG00000211630 | IGKV1D-13 | 1.610126556 | 0.093714318 | -0.264093563 | 0.587695191 | -0.638143693 | 0.163382764 | -0.479857165 | 0.371937803 | 0.386905523 | 0.000348924 | 0.089148818 | 0.771618424 | 0.263995413 | 0.340662558 | positive |
| ENSG00000211933 | IGHV6-1 | 2.796109915 | 6.9486E-06 | -0.880401725 | 0.00314506 | -0.916928436 | 0.00109341 | -0.741326315 | 0.021043207 | 0.380067716 | 5.66513E-08 | -0.247135234 | 0.171281542 | 0.58307469 | 0.626504409 | positive |
| ENSG00000211660 | IGLV2-23 | 3.585212436 | 2.70147E-06 | -0.948773111 | 0.008095728 | -0.937981718 | 0.00513429 | -0.240532968 | 0.525595599 | 0.373838411 | 3.63721E-06 | 0.133631357 | 0.5388316 | 0.368182523 | 0.433996843 | positive |
| ENSG00000167236 | CCL23 | 0.368891381 | 0.725929453 | 0.216355562 | 0.689598579 | 0.274315924 | 0.5867355 | 1.728795498 | 0.005543193 | 0.373329053 | 0.001607243 | -0.802524223 | 0.022745003 | 0.436162074 | 0.494895191 | positive |
| ENSG00000239855 | IGKV1-6 | 2.719470156 | 6.58943E-05 | -0.54457935 | 0.092653665 | -0.61520935 | 0.042833389 | 0.373524591 | 0.289571948 | 0.372936413 | 9.43829E-07 | 0.030992475 | 0.877518813 | 0.44351951 | 0.501486228 | positive |
| ENSG00000211955 | IGHV3-33 | 4.390059494 | 1.28761E-09 | -0.99756724 | 0.001475275 | -1.038694588 | 0.000450121 | -0.273546616 | 0.401753887 | 0.372521643 | 2.1539E-07 | -0.16738276 | 0.371564204 | 0.525935461 | 0.575317184 | positive |
| ENSG00000254140 | RP11-731F5.1 | -0.579890715 | 0.287647573 | 0.414639475 | 0.142003998 | 0.143515359 | 0.580965456 | 0.38991702 | 0.20788184 | 0.368100354 | 8.22351E-08 | -0.131881354 | 0.455194831 | 0.654358096 | 0.690362461 | positive |
| ENSG00000224041 | IGKV3D-15 | 4.465043496 | 7.93546E-10 | -0.761874742 | 0.012722073 | -0.779815711 | 0.006529357 | -0.383907318 | 0.240194849 | 0.36660762 | 2.8668E-07 | -0.14372973 | 0.44142734 | 0.506739902 | 0.558121163 | positive |
| ENSG00000175352 | NRIP3 | 1.447106161 | 0.096496598 | 0.236444019 | 0.592340307 | 0.476395328 | 0.24936758 | 0.829471755 | 0.092561354 | 0.364479904 | 0.000219165 | -0.532539256 | 0.060909219 | 0.489161216 | 0.54237359 | positive |
| ENSG00000240671 | IGKV1-8 | 2.759857184 | 0.000379273 | -0.305586262 | 0.411225498 | -0.337316809 | 0.330614313 | -0.364602334 | 0.373141235 | 0.358815812 | 2.44479E-05 | -0.250325412 | 0.287608986 | 0.426944169 | 0.486637485 | positive |
| ENSG00000244437 | IGKV3-15 | 5.473023709 | 1.20615E-12 | -0.702867209 | 0.017645186 | -0.707600445 | 0.010687001 | -0.265810315 | 0.400916682 | 0.35784093 | 2.62997E-07 | -0.210846923 | 0.247384013 | 0.525975046 | 0.575352645 | positive |
| ENSG00000239951 | IGKV3-20 | 6.521566264 | 6.03414E-14 | -0.532766823 | 0.090952965 | -0.662284226 | 0.025802757 | -0.205632866 | 0.54725301 | 0.356294663 | 1.33387E-06 | -0.2131776 | 0.279200122 | 0.476670008 | 0.531183549 | positive |
| ENSG00000211685 | IGLC7 | 1.890706615 | 0.00901731 | -0.546575305 | 0.131746863 | -0.575511107 | 0.089372001 | 0.158372221 | 0.687711014 | 0.354163894 | 1.75024E-05 | 0.035533136 | 0.875024475 | 0.34632258 | 0.414413978 | positive |
| ENSG00000240864 | IGKV1-16 | 4.079998832 | 1.1687E-07 | -0.914770417 | 0.008412406 | -1.099472485 | 0.000902872 | -0.416920328 | 0.258804206 | 0.349374753 | 6.81886E-06 | 0.02869329 | 0.891483647 | 0.374683052 | 0.439820234 | positive |
| ENSG00000211947 | IGHV3-21 | 4.32914848 | 3.25489E-10 | -0.82208856 | 0.004546061 | -0.649760292 | 0.014687883 | -0.219728532 | 0.470842905 | 0.349085275 | 2.05793E-07 | -0.062636111 | 0.719579485 | 0.51241201 | 0.563202426 | positive |
| ENSG00000207425 | Y_RNA | 1.096306433 | 0.410702567 | -0.258646916 | 0.705202193 | -0.907467118 | 0.158408675 | -0.328700386 | 0.662183165 | 0.348572972 | 0.016674405 | -0.24012893 | 0.578356268 | 0.112270051 | 0.204741921 | positive |
| ENSG00000211959 | IGHV4-39 | 4.431896659 | 1.43492E-09 | -0.797973343 | 0.010378365 | -0.659599815 | 0.021801514 | -0.446660401 | 0.179695559 | 0.348062527 | 1.08401E-06 | -0.016250861 | 0.931494311 | 0.460661345 | 0.516842454 | positive |
| ENSG00000243466 | IGKV1-5 | 6.145163006 | 4.22805E-12 | -0.599570445 | 0.079261371 | -0.511230787 | 0.106930167 | 0.075143867 | 0.838698914 | 0.344147526 | 9.85945E-06 | -0.113002686 | 0.594270885 | 0.404685972 | 0.46669785 | positive |
| ENSG00000220267 | ACTBP8 | 0.441258597 | 0.814657677 | 0.49703191 | 0.608340108 | -0.608689855 | 0.500681318 | 0.612247611 | 0.566258239 | 0.343923403 | 0.090129982 | 1.040135865 | 0.094252975 | 0.113339777 | 0.205700217 | positive |
| ENSG00000211668 | IGLV2-11 | 3.721593089 | 4.27915E-06 | -0.852182708 | 0.02393449 | -0.84964104 | 0.016017785 | 0.007061497 | 0.986012631 | 0.341669901 | 4.27432E-05 | 0.062902122 | 0.785616032 | 0.29896208 | 0.371986863 | positive |
| ENSG00000164742 | ADCY1 | -0.848575657 | 0.293882854 | 0.011427996 | 0.977945058 | 0.179537867 | 0.641228496 | 0.270598151 | 0.552641195 | 0.336417937 | 0.000266234 | -0.449766811 | 0.090179485 | 0.430772063 | 0.49006664 | positive |
| ENSG00000211640 | IGLV6-57 | 3.891241616 | 1.10308E-06 | -0.540355942 | 0.133385164 | -0.50517123 | 0.131759194 | 0.178195732 | 0.64883786 | 0.335714772 | 3.55439E-05 | 0.010206942 | 0.963703395 | 0.331922016 | 0.401513472 | positive |
| ENSG00000211666 | IGLV2-14 | 5.2775796 | 2.54526E-09 | -0.707444432 | 0.057667952 | -0.713798763 | 0.040348039 | -0.016237778 | 0.967728851 | 0.332972279 | 5.88494E-05 | -0.196975128 | 0.39446087 | 0.352863729 | 0.420273758 | positive |
| ENSG00000211946 | IGHV3-20 | 1.694953378 | 0.051068611 | -1.282792892 | 0.005105877 | -1.037660566 | 0.013900894 | -0.682686674 | 0.160585054 | 0.331693307 | 0.000600984 | 0.272720003 | 0.325843759 | 0.20996764 | 0.292262678 | positive |
| ENSG00000211964 | IGHV3-48 | 3.792619748 | 6.19714E-08 | -0.727951806 | 0.01910265 | -0.637654223 | 0.026887455 | 0.097962105 | 0.767215697 | 0.330663004 | 2.99932E-06 | 0.061724765 | 0.74519582 | 0.397719624 | 0.460457164 | positive |
| ENSG00000241351 | IGKV3-11 | 6.141515085 | 8.16587E-16 | -0.657146889 | 0.013317876 | -0.755695086 | 0.002659339 | -0.440658964 | 0.122872415 | 0.327991923 | 1.55815E-07 | -0.124934287 | 0.441120492 | 0.521297174 | 0.571162051 | positive |
| ENSG00000105974 | CAV1 | 1.120169743 | 0.016183755 | -0.475717702 | 0.044921897 | -0.310855374 | 0.154304297 | 0.029216793 | 0.908728098 | 0.327264846 | 1.81333E-08 | -0.07750054 | 0.596749679 | 0.598745242 | 0.640542613 | positive |
| ENSG00000161905 | ALOX15 | 6.346061279 | 7.50226E-07 | -0.211924909 | 0.709216793 | -0.111586871 | 0.832847341 | 0.533397289 | 0.39518606 | 0.324244072 | 0.007874047 | -1.086342181 | 0.003930502 | 0.375895091 | 0.440906019 | positive |
| ENSG00000211598 | IGKV4-1 | 6.529535816 | 4.39501E-14 | -0.870038568 | 0.006712075 | -0.801131913 | 0.007277987 | -0.108321663 | 0.748726971 | 0.321881413 | 6.93957E-06 | 0.030426507 | 0.875358632 | 0.373272524 | 0.438556636 | positive |
| ENSG00000211671 | IGLV2-8 | 4.192855898 | 2.7501E-06 | -0.593447958 | 0.145165521 | -0.863193524 | 0.02524478 | -0.158869615 | 0.719816198 | 0.320646847 | 0.000341924 | -0.181623798 | 0.475853415 | 0.277489216 | 0.352750756 | positive |
| ENSG00000048462 | TNFRSF17 | 4.073473242 | 2.48655E-06 | -0.732219286 | 0.064831099 | -0.634798566 | 0.084686986 | -0.195585437 | 0.647751263 | 0.319934258 | 0.000231353 | -0.034324141 | 0.888759204 | 0.265151996 | 0.341698663 | positive |
| ENSG00000231475 | IGHV4-31 | 3.148776192 | 0.001498328 | -0.627963304 | 0.195654495 | -0.960362615 | 0.036426877 | -0.568074986 | 0.285760586 | 0.318791474 | 0.002316386 | -0.267491652 | 0.380062624 | 0.224726208 | 0.305483895 | positive |
| ENSG00000211966 | IGHV5-51 | 4.721606925 | 4.75078E-10 | -0.800539363 | 0.011642361 | -0.52104121 | 0.072284611 | -0.159249372 | 0.636104387 | 0.318354526 | 7.61892E-06 | -0.159866353 | 0.409112111 | 0.45147441 | 0.508612492 | positive |
| ENSG00000170866 | LILRA3 | 3.49379516 | 0.223873197 | -0.882817944 | 0.547760931 | -0.806243595 | 0.555325898 | -0.366576235 | 0.820185939 | 0.318019898 | 0.295337756 | 0.214401097 | 0.816802252 | -0.0839616 | 0.028951067 | positive |
| ENSG00000202538 | RNU4-2 | 4.245617523 | 0.000318847 | 1.426444859 | 0.014232192 | 1.585450195 | 0.003898645 | 0.010288904 | 0.986710629 | -0.338556737 | 0.005214068 | -0.764045881 | 0.035745338 | 0.116518875 | 0.208548159 | negative |
| ENSG00000248923 | MTND5P11 | 4.192452607 | 0.001568816 | 1.12882531 | 0.084445467 | 1.586610809 | 0.01074408 | 0.0635306 | 0.928419156 | -0.33014532 | 0.016022994 | -0.605413732 | 0.140790967 | 0.072785936 | 0.169370734 | negative |
| ENSG00000206588 | RNU1-8 | 8.174816697 | 9.26755E-09 | 1.756326184 | 0.004943902 | 1.579969043 | 0.006447564 | 0.521474909 | 0.428226042 | -0.305604231 | 0.016206632 | -0.917994736 | 0.018280449 | 0.100497458 | 0.194195639 | negative |
| ENSG00000207418 | RNU1-9 | 8.101732933 | 9.64023E-09 | 1.726703613 | 0.005338382 | 1.5518188 | 0.006985243 | 0.48499884 | 0.45775102 | -0.30168441 | 0.016771949 | -0.907169988 | 0.018781561 | 0.097593514 | 0.19159419 | negative |
| ENSG00000206585 | RNU1-6 | 8.097444631 | 1.00545E-08 | 1.72386438 | 0.005461183 | 1.54811574 | 0.007176911 | 0.470028825 | 0.472215591 | -0.301206341 | 0.01706439 | -0.905431666 | 0.019136901 | 0.096482664 | 0.190599053 | negative |
| ENSG00000206652 | RNU1-1 | 8.164322932 | 6.00873E-09 | 1.702910474 | 0.005410407 | 1.526148078 | 0.00722321 | 0.486283779 | 0.451111281 | -0.299529197 | 0.016266655 | -0.896770965 | 0.01871072 | 0.097575348 | 0.191577916 | negative |
| ENSG00000207513 | RNU1-3 | 8.19906309 | 4.46495E-09 | 1.670390582 | 0.005903414 | 1.508555227 | 0.007414146 | 0.475784918 | 0.457284231 | -0.299418459 | 0.015511678 | -0.892692786 | 0.018325589 | 0.096689749 | 0.190784567 | negative |
| ENSG00000206737 | RNU1-5 | 8.150986731 | 6.61431E-09 | 1.713099391 | 0.005259776 | 1.524000366 | 0.007427431 | 0.465958537 | 0.471178792 | -0.298715584 | 0.016781585 | -0.895144956 | 0.019179246 | 0.097132454 | 0.191181157 | negative |
| ENSG00000207389 | RNU1-4 | 8.190107093 | 4.67789E-09 | 1.660847862 | 0.006207777 | 1.505693052 | 0.007564126 | 0.47050833 | 0.462625989 | -0.298184451 | 0.015995553 | -0.893210147 | 0.01835284 | 0.095236625 | 0.18948281 | negative |
| ENSG00000206596 | RNU1-7 | 8.083935458 | 7.68734E-09 | 1.716174477 | 0.005106131 | 1.540488374 | 0.006757989 | 0.478335598 | 0.458679938 | -0.295811474 | 0.017594924 | -0.894957051 | 0.018982517 | 0.097977988 | 0.191938614 | negative |
| ENSG00000207005 | RNU1-2 | 8.134192604 | 6.06284E-09 | 1.656866767 | 0.00651212 | 1.49458079 | 0.008211894 | 0.437607895 | 0.495992229 | -0.292630494 | 0.018356473 | -0.903275951 | 0.017567411 | 0.092554865 | 0.1870804 | negative |
| ENSG00000134321 | RSAD2 | 12.50423714 | 9.70549E-15 | 1.080171611 | 0.05958589 | 1.992656736 | 0.000405728 | 1.527157823 | 0.016850423 | -0.274889924 | 0.021298067 | -1.513330815 | 9.81288E-05 | 0.371648679 | 0.437101942 | negative |
| ENSG00000264462 | MIR3648 | 6.08403497 | 1.52009E-07 | 1.654645574 | 0.0018993 | 1.61400229 | 0.001205164 | 1.073645364 | 0.057495326 | -0.253046842 | 0.018154318 | -0.771765498 | 0.018666776 | 0.156576446 | 0.244433066 | negative |
| ENSG00000254415 | SIGLEC14 | 12.16985958 | 1.23706E-11 | 0.937398281 | 0.17852371 | 0.642075097 | 0.319871007 | -0.908172209 | 0.235002127 | -0.251459026 | 0.082029528 | -0.09421199 | 0.828672402 | 0.041831595 | 0.141640804 | negative |
| ENSG00000239776 | AC079949.1 | 7.381496355 | 3.90167E-07 | 1.912482242 | 0.004348811 | 2.099477325 | 0.000945691 | 1.993666428 | 0.00663308 | -0.24917447 | 0.063621181 | -0.545937166 | 0.180483434 | 0.191438213 | 0.275663399 | negative |
| ENSG00000137959 | IFI44L | 11.47937247 | 8.36905E-15 | 0.614490307 | 0.234985407 | 1.42191893 | 0.004549283 | 1.014080084 | 0.07778102 | -0.247445335 | 0.023202798 | -1.284979485 | 0.000252986 | 0.328893316 | 0.398800262 | negative |
| ENSG00000110203 | FOLR3 | 11.99989316 | 3.23926E-13 | 1.739375912 | 0.005733109 | 1.225867217 | 0.033098367 | 0.141490264 | 0.830760081 | -0.242674343 | 0.0553923 | -0.185016094 | 0.626648772 | 0.123892172 | 0.215153404 | negative |
| ENSG00000255860 | RP11-807H22.6 | 5.848669686 | 1.07984E-06 | 1.605372683 | 0.004158504 | 1.228296563 | 0.016789309 | 0.318506392 | 0.587910877 | -0.238793201 | 0.034339822 | -0.289575866 | 0.391864783 | 0.113789058 | 0.206102698 | negative |
| ENSG00000165949 | IFI27 | 7.58920287 | 4.56423E-08 | 1.835940274 | 0.003295218 | 1.745488923 | 0.002741055 | 1.445554039 | 0.031198219 | -0.234198244 | 0.060612186 | -1.088065607 | 0.005529177 | 0.167945419 | 0.254617771 | negative |
| ENSG00000138759 | FRAS1 | 3.349923089 | 0.03745007 | 1.225071846 | 0.134374821 | 1.503440354 | 0.050554473 | 0.04371759 | 0.960753446 | -0.233367246 | 0.165512909 | -0.759761388 | 0.1412073 | -0.009036741 | 0.096071253 | negative |
| ENSG00000151468 | CCDC3 | 4.730794867 | 1.67765E-10 | 1.078793866 | 0.000633121 | 0.942275198 | 0.001234986 | 0.000701113 | 0.998272394 | -0.232623682 | 0.000377781 | -0.37293808 | 0.04978767 | 0.228094786 | 0.308501579 | negative |
| ENSG00000241781 | AL161626.1 | 9.914734356 | 3.77873E-11 | 1.298040294 | 0.030682927 | 1.578049619 | 0.005573968 | 1.404922451 | 0.033331014 | -0.230351193 | 0.060881317 | -0.500307863 | 0.179555487 | 0.102517414 | 0.196005184 | negative |
| ENSG00000137965 | IFI44 | 12.36657954 | 4.22315E-19 | 0.532600009 | 0.204613273 | 1.177117747 | 0.003822328 | 0.771151534 | 0.097252627 | -0.225204408 | 0.01140444 | -0.991176603 | 0.00045473 | 0.309687343 | 0.381594911 | negative |
| ENSG00000185745 | IFIT1 | 13.00230088 | 1.52078E-17 | 0.679215247 | 0.164511989 | 1.460395926 | 0.002142979 | 1.322990613 | 0.016157262 | -0.221205283 | 0.030636564 | -1.023394236 | 0.001573355 | 0.306222773 | 0.378491234 | negative |
| ENSG00000205837 | LINC00487 | 5.423043951 | 2.23343E-07 | 0.763678933 | 0.099816198 | 1.223715959 | 0.005928824 | 0.926626437 | 0.070432087 | -0.217103863 | 0.025103191 | -0.99325457 | 0.001213431 | 0.231766576 | 0.311790891 | negative |
| ENSG00000169429 | IL8 | 9.238090129 | 5.63344E-13 | 0.746733907 | 0.118536801 | 1.029639913 | 0.022919865 | -0.008326322 | 0.98719428 | -0.205829347 | 0.038857192 | -0.220297853 | 0.460743849 | 0.048261456 | 0.147400888 | negative |
| ENSG00000200488 | 7SK | 3.852549009 | 2.65724E-05 | 0.933295404 | 0.03218426 | 0.97383858 | 0.017060837 | 0.561024427 | 0.232979118 | -0.200865423 | 0.025589731 | -0.62201231 | 0.024152899 | 0.071524269 | 0.168240491 | negative |
| ENSG00000213934 | HBG1 | 13.51991896 | 5.75577E-12 | 1.066105438 | 0.15777949 | 0.467938989 | 0.501484809 | 0.489840341 | 0.551368165 | -0.200306539 | 0.196814206 | 0.522280596 | 0.270801033 | 0.086138391 | 0.181332308 | negative |
| ENSG00000265735 | Metazoa_SRP | 9.191149604 | 9.16879E-15 | 1.589719038 | 0.000352543 | 1.402005259 | 0.000651276 | 0.966908042 | 0.037579475 | -0.199058097 | 0.022953117 | -0.212866143 | 0.414957512 | 0.241681779 | 0.32067326 | negative |
| ENSG00000134326 | CMPK2 | 10.84060318 | 4.11374E-17 | 0.722948116 | 0.086374233 | 1.433012935 | 0.000544164 | 1.107176898 | 0.018736911 | -0.198996726 | 0.023727408 | -1.094365117 | 0.0001285 | 0.371538446 | 0.437003191 | negative |
| ENSG00000154589 | LY96 | 10.7260371 | 3.04297E-21 | 0.701396628 | 0.030814785 | 0.94820425 | 0.002290895 | 0.324001144 | 0.35368888 | -0.19814048 | 0.003787766 | 0.258041835 | 0.200121648 | 0.324135171 | 0.394537757 | negative |
| ENSG00000229390 | MICD | 7.516932593 | 2.13421E-09 | 0.434299848 | 0.401887493 | 0.771841286 | 0.113323558 | -0.288825999 | 0.611402854 | -0.197457813 | 0.068544761 | -0.014188498 | 0.965257243 | 0.029258124 | 0.130377069 | negative |
| ENSG00000111331 | OAS3 | 13.40834318 | 9.08974E-20 | 0.65613473 | 0.134338321 | 1.273712356 | 0.00271375 | 0.767145839 | 0.112154887 | -0.193214631 | 0.034698079 | -1.172862259 | 9.02419E-05 | 0.331480233 | 0.401117709 | negative |
| ENSG00000088827 | SIGLEC1 | 10.0894397 | 4.72762E-15 | 0.700019634 | 0.119501062 | 1.320580808 | 0.002447383 | 0.809983025 | 0.102029469 | -0.192100684 | 0.040193732 | -1.23303677 | 6.34247E-05 | 0.340130856 | 0.408867226 | negative |
| ENSG00000243064 | ABCC13 | 7.090890787 | 4.47992E-10 | 1.587960407 | 0.001133544 | 1.157630162 | 0.009092685 | 1.180554091 | 0.023013681 | -0.189832092 | 0.049190775 | 0.117132436 | 0.685582802 | 0.265929751 | 0.342395402 | negative |
| ENSG00000157168 | NRG1 | 9.705651805 | 4.32503E-15 | 0.787298708 | 0.069417208 | 0.652730824 | 0.104479888 | -0.133708951 | 0.775122373 | -0.1872661 | 0.037233744 | -0.357022705 | 0.188053047 | 0.014030963 | 0.116736071 | negative |
| ENSG00000256049 | PADI6 | 5.989816265 | 1.25212E-09 | 0.940377428 | 0.023435374 | 1.012079901 | 0.009454398 | -0.033285028 | 0.940080913 | -0.186154459 | 0.02908651 | -0.505803363 | 0.051605756 | 0.07023828 | 0.167088459 | negative |
| ENSG00000012223 | LTF | 11.99926421 | 2.22837E-09 | 0.877256342 | 0.290830221 | 0.723631442 | 0.348580817 | 1.986714949 | 0.03310759 | -0.185440441 | 0.278637543 | 0.328466729 | 0.529261021 | 0.054630469 | 0.153106462 | negative |
| ENSG00000133742 | CA1 | 9.404735777 | 2.05639E-11 | 1.591288903 | 0.005107213 | 1.086976086 | 0.035927746 | 1.261740026 | 0.039185144 | -0.184523121 | 0.103985147 | 0.691995852 | 0.048268245 | 0.351497725 | 0.419050046 | negative |
| ENSG00000199916 | RMRP | 4.851040098 | 3.91822E-06 | 0.876047148 | 0.070366256 | 0.907473994 | 0.044987593 | 0.744333825 | 0.159035627 | -0.184389096 | 0.064734165 | -0.824318798 | 0.00833529 | 0.097577707 | 0.191580029 | negative |
| ENSG00000206177 | HBM | 10.52968167 | 1.08282E-15 | 2.129850196 | 1.70625E-05 | 1.571589466 | 0.000403353 | 1.825914598 | 0.000490507 | -0.183770634 | 0.048897033 | -0.245615202 | 0.38155471 | 0.373899329 | 0.439118149 | negative |
| ENSG00000197846 | HIST1H2BF | 5.913179738 | 6.65761E-17 | 0.351875564 | 0.129771186 | 0.616260306 | 0.005763328 | 0.04647873 | 0.853740833 | -0.183718912 | 0.000318677 | 0.359102906 | 0.016438946 | 0.456991984 | 0.513555319 | negative |
| ENSG00000245848 | CEBPA | 7.065847666 | 1.34181E-08 | 1.279281627 | 0.018070636 | 1.173646961 | 0.019693234 | 1.315335179 | 0.026523142 | -0.183526404 | 0.09417347 | -0.259754389 | 0.433519615 | 0.088393784 | 0.183352764 | negative |
| ENSG00000109272 | PF4V1 | 7.175870635 | 1.29084E-12 | 1.750580872 | 2.88878E-05 | 1.521573107 | 7.78113E-05 | 0.946411584 | 0.026445706 | -0.182555895 | 0.022476648 | -0.437681213 | 0.070836855 | 0.300896289 | 0.373719592 | negative |
| ENSG00000167914 | GSDMA | 5.200029762 | 9.20761E-10 | 0.49980818 | 0.152606851 | 0.660341594 | 0.044810033 | -0.184520833 | 0.627552325 | -0.182375844 | 0.013414817 | -0.160650188 | 0.462492145 | 0.065127875 | 0.162510388 | negative |
| ENSG00000185044 | BX004987.4 | 6.529182675 | 1.38487E-12 | 0.538508037 | 0.121961833 | 0.645679665 | 0.048340304 | -0.236385646 | 0.532326931 | -0.180183771 | 0.013986749 | -0.55619436 | 0.01334185 | 0.094579668 | 0.188894286 | negative |
| ENSG00000238103 | RPL9P7 | 10.50595845 | 3.23012E-15 | 0.477270414 | 0.298623601 | 0.475844211 | 0.266043209 | 0.023492226 | 0.962671454 | -0.180103497 | 0.060601512 | -0.195864338 | 0.497696748 | -0.025248192 | 0.081548495 | negative |
| ENSG00000100985 | MMP9 | 12.64618697 | 7.53015E-20 | 0.659157618 | 0.109626813 | 0.725224627 | 0.060102459 | 0.563823099 | 0.21082638 | -0.17869183 | 0.03725965 | 0.855281325 | 0.001653865 | 0.410090172 | 0.471539113 | negative |
| ENSG00000230903 | RPL9P8 | 12.96274699 | 2.72007E-17 | 0.40177816 | 0.413721969 | 0.419483058 | 0.359750965 | -0.045961736 | 0.932002731 | -0.178216171 | 0.082654311 | -0.094130503 | 0.760859307 | -0.031328762 | 0.076101318 | negative |
| ENSG00000253549 | RP11-317J10.2 | 4.963438964 | 2.34793E-11 | 1.093683208 | 0.000417282 | 0.778090522 | 0.005498451 | 0.614733408 | 0.057160202 | -0.178192431 | 0.004183214 | -0.15647459 | 0.390794063 | 0.239789606 | 0.318978189 | negative |
|  |  |  |  |  |  |  |  |  |  |  |  |  |  |  |  |  |

| **Supplementary Table 3. Genes used in gene signatures** | |
| --- | --- |
| **Gene Signature** | **Genes in the Signature** |
| B cell | BANK1 BLNK CD22 CD79A EBF1 FCRL1 FCRLA MS4A1 TCL1A VPREB3 |
| EOSINOPHIL | TKTL1 CD9 PRSS33 CLC CAT LGALS12 CAMK1 SIGLEC10 FLVCR1 EMR1 P2RY14 CAMK1D CCR3 FAM101B OLIG1 VSTM1 |
| NEUTROPHIL | DGAT2 PYGL C20orf3 BMX AQP9 IL1R2 FAM129A WDFY3 CA4 LRG1 KREMEN1 CR1 PLIN5 |
| Th1 | TBX21 IFNG CXCR3 |
| Th2 | GATA3 IL4 IL13 CCR4 |
| Treg | FOXP3 TGFB1 IL10 TNFRSF18 |
| Tfh | BCL6 CXCR5 ICOS SH2D1A PDCD1 |
| IL4 | IL11 PF4V1 RGS5 CXCL3 APOBEC3B SLC22A4 |
| IL13 | MAP2K6 WNT5B PHACTR1 CISH CTNNAL1 MRC1 EGR2 HRH4 ANKRD22 DACH1 MAOA SUCNR1 TGM2 |
